# Stabilized Double-Stranded RNA Strategy Improves Cotton Resistance to CBW (*Anthonomus grandis*)

**DOI:** 10.3390/ijms232213713

**Published:** 2022-11-08

**Authors:** Thuanne P. Ribeiro, Daniel D. N. Vasquez, Leonardo L. P. Macedo, Isabela T. Lourenço-Tessutti, David C. Valença, Osmundo B. Oliveira-Neto, Bruno Paes-de-Melo, Paolo L. Rodrigues-Silva, Alexandre A. P. Firmino, Marcos F. Basso, Camila B. J. Lins, Maysa R. Neves, Stefanie M. Moura, Bruna M. D. Tripode, José E. Miranda, Maria C. M. Silva, Maria F. Grossi-de-Sa

**Affiliations:** 1 Embrapa Genetic Resources and Biotechnology, Brasilia 70770-917, DF, Brazil; 2 Biotechnology and Molecular Biology Department, Federal University of Brasilia (UnB), Brasilia 70910-900, DF, Brazil; 3 Genetic and Molecular Biology Department, Catholic University of Brasilia (UCB), Brasilia 71966-700, DF, Brazil; 4 National Institute of Science and Technology (INCT Plant Stress Biotech), Embrapa, Brasilia 70770-917, DF, Brazil; 5 Biochemistry and Molecular Biology Department, Integrated Faculties of the Educational Union of Planalto Central, Brasilia 70675-760, DF, Brazil; 6 Max Planck Institute Molecular Plant Physiol, 14476 Potsdam, Germany; 7 Embrapa Cotton, Goiânia 74605-170, GO, Brazil

**Keywords:** RNA interference, *Gossypium hirsutum*, *Anthonomus grandis*, gene silencing, knockdown, viroid genome

## Abstract

Cotton is the most important crop for fiber production worldwide. However, the cotton boll weevil (CBW) is an insect pest that causes significant economic losses in infested areas. Current control methods are costly, inefficient, and environmentally hazardous. Herein, we generated transgenic cotton lines expressing double-stranded RNA (dsRNA) molecules to trigger RNA interference-mediated gene silencing in CBW. Thus, we targeted three essential genes coding for chitin synthase 2, vitellogenin, and ecdysis-triggering hormone receptor. The stability of expressed dsRNAs was improved by designing a structured RNA based on a viroid genome architecture. We transformed cotton embryos by inserting a promoter-driven expression cassette that overexpressed the dsRNA into flower buds. The transgenic cotton plants were characterized, and positive PCR transformed events were detected with an average heritability of 80%. Expression of dsRNAs was confirmed in floral buds by RT-qPCR, and the T_1_ cotton plant generation was challenged with fertilized CBW females. After 30 days, data showed high mortality (around 70%) in oviposited yolks. In adult insects fed on transgenic lines, chitin synthase II and vitellogenin showed reduced expression in larvae and adults, respectively. Developmental delays and abnormalities were also observed in these individuals. Our data remark on the potential of transgenic cotton based on a viroid-structured dsRNA to control CBW.

## 1. Introduction

Cotton (*Gossypium hirsutum*) is the world’s most important fiber crop, estimated at $600 billion per year. One of the major challenges in cotton production is the control of insect pests [1]. Brazil is among the largest cotton producers in the world; however, several crop areas are severely affected by the cotton boll weevil (CBW, *Anthonomus grandis*), a devastating insect pest that could reduce cotton yield by 100% if left uncontrolled [2]. The use of chemical insecticides remains widespread in the control of this pest. Nevertheless, it is an expensive procedure with limited efficacy due to the endophytic nature of CBW larvae [3].

As an alternative to pesticides, genetically modified (GM) crops have been widely used for insect pest control over the past two decades. Notwithstanding, there are no commercial transgenic cotton events capable of controlling CBW. In recent years, transgenic cotton studies have been reported showing improved resistance to CBW through overexpression of Cry toxin [4,5]. Efforts have been made for other alternative technologies, as resistance traits can rapidly be observed in insect pest populations feeding on currently available GM crops [6,7].

RNA interference (RNAi) is a molecular mechanism that plays an important role in regulating gene expression in eukaryotic cells. Through complex enzymatic machinery and different pathways, it is possible to effectively reduce gene expression. With the appropriate method, exogenous delivery of double-stranded RNA (dsRNA) molecules into a cell, a pathway known as post-transcriptional silencing mediated by small-interfering RNA (siRNA), can knockdown a target gene in a highly specific manner [8]. Due to their unique specificity and efficacy, dsRNA molecules are being developed and exploited as a biotechnology tool for controlling insect pests by silencing genes considered essential for insect pest survival [7,9,10,11]. As for the practical application of RNAi for pest control, transgenic plants expressing dsRNA have been developed and applied to achieve resistance to coleopteran pests in a series of research over the last 10 years [12,13,14,15,16].

Currently, Brazil is one of the world’s leading countries in RNAi research for crop protection [7]. Remarkable advances have been made by different groups in developing transgenic and non-transgenic approaches to improve plant resistance to a range of pests [17,18,19,20,21]. Some of the contributions worth highlighting are the discovery and validation of potential target genes to design RNAi molecules to control the CBW [22,23,24,25], the improvement of the understanding of basic mechanisms of RNAi in insects [11], and the design of novel approaches to improve RNAi efficacy [26].

In spite of these advances, using RNAi to control CBW remains challenging. Although RNAi is robust in most coleopterans [27,28], CBW exhibits reduced efficiency in experiments when dsRNA molecules are ingested by larvae or adult insects [29]. Another factor also contributes to the low efficacy of GM plants’ delivery systems. Plants have all the elements to trigger RNAi responses; as a result, dsRNA molecules can be cut into siRNA fragments within the plant’s cells and interrupt their processing in the target pest, resulting in little or no RNAi response [30]. Non-linear RNAs have been successfully used to improve the effect of RNAi against insects in plant-mediated systems [31]. Interestingly, some plant pathogens, such as viroids, exhibit an RNA secondary structure that persists in plant tissues and cells without being degraded [32]. Viroid structures to enhance dsRNA delivery are currently being used to obtain transgenic plants [33]. A viroid-structured dsRNA would no longer be available in either the RNAi machinery in the plant nor in the target insect. However, in conjunction with an endonucleolytic ribozyme (capable of self-cleavage at specific pHs), it is possible to exploit the pH differences between the extracellular environment of the host and the insect. Therefore, the disassembly of the viroid structure is triggered only in the digestive system of the target insect [34,35].

In addition to the stability of dsRNA molecules, a crucial factor affecting RNAi efficiency in insect pests is the selection of target genes. High specificity and lethality are undeniable requirements; however, temporal and spatial expression patterns, as well as the half-life of encoded proteins, must also be considered before selecting a target gene [36]. For example, two previous studies reported high mortality rates in *A. grandis* (larvae and adult insects) microinjected with low doses of dsRNA targeting the chitin synthase II (*AgCHS2*) and vitellogenin (*AgVg*) genes [37,38].

**Figure 1:**
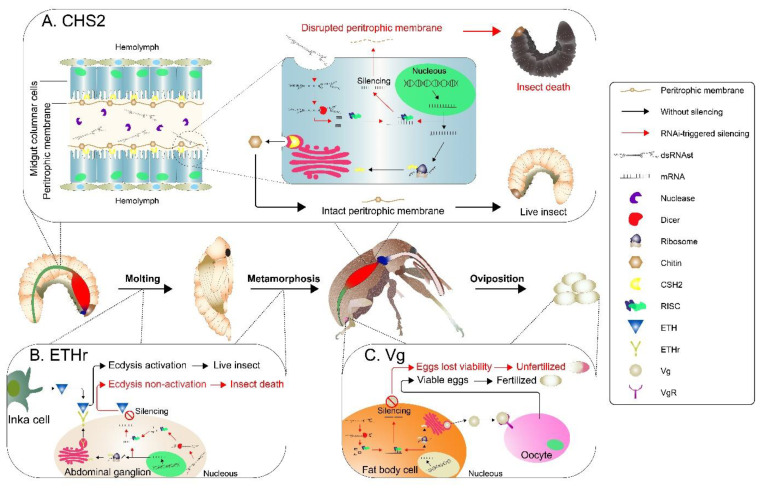
Schematic representation of the physiological and phenotypic effects of RNAi-mediated knockdown of chitin synthase II (*CHS2*), vitellogenin (*Vg*), and Ecdysis-triggering hormone receptor (*ETHr*) in *A. grandis*. After feeding on transgenic cotton buds, the larva ingests structured dsRNA molecules (dsRNAst). The dsRNAst will be internalized by midgut columnar cells. Red arrows indicate the RNAi-mediated knockdown of target genes and its consequences on insect survival. In the presence of dsRNAst, DICER proteins convert dsRNA molecules into siRNA fragments that bind to the RNA-induced silencing complex (RISC) to degrade the target gene mRNA and, thus, prevent protein translation. Black arrows indicate the function and physiological cascades of the target genes with their endogenous gene expression (w/o RNAi). The *A. grandis* gut is divided into anterior midgut (blue), posterior midgut (red), and hindgut (green). (**A**) CHS2 is produced at all life stages of *A. grandis* and participates in the biosynthesis of chitin, which composes the peritrophic membrane (PM), allowing nutrient absorption. When interrupted by RNAi, the reconstruction of PM fails. As a result, absorption rates in the gut cells decrease, leading to nutritional deficiency and death. (**B**) Ecdysis triggers molting and metamorphosis processes in insects. Ecdysis-triggering hormones (ETHs) are released from endocrine Inka cells and initiate the ecdysis-signaling pathway on central neurons or the abdominal ganglion by binding to ETHr. The *ETHr* silencing interrupts ecdysis cascades at the intracellular level, causing developmental delay, abnormal pupation, and death. (**C**) Vg protein, involved in yolk formation, is synthesized in fat bodies. Then, it is secreted into the hemolymph and transported through the circulatory system to the ovary, where it is internalized into the oocytes through binding to the Vg receptor (VgR) and stored in the yolk. The embryo uses this component as a primary food source within the eggs. If Vg production is decreased by gene silencing, it will prevent complete egg formation and generate non-viable embryos.

The *CHS2* gene encodes an intestinal protein that is unique to arthropods and fungi. It is involved in the synthesis of chitin, a polysaccharide essential for peritrophic membrane (PM) [39]. *CHS2* deficiency leads to disruption of the PM, which significantly impairs nutrient absorption as well as intestinal immunity (Figure 1A). For this reason, several commercial insecticides are inhibitors of chitin synthesis [40]. Regardless, due to their lack of specificity, biotechnological alternatives have been explored in recent years. For instance, RNAi-mediated knockdown of *CHS2* has shown promising results in several insect species [41,42,43,44,45]. On the other hand, the *Vg* gene encodes an egg yolk precursor present in the females of most oviparous species. In insects, Vg is essential for egg viability (Figure 1B), providing a food source for embryo development [46,47]. Different studies have shown that silencing of the *Vg* gene in insects can reduce the fitness of the offspring of a treated female. This effect is known as a parental-RNAi effect, a desirable feature for biotechnological products focused on insect pest control [48,49,50,51,52]. In addition, genes related to hormonal regulation are generally effective in triggering lethal phenotypes in RNAi experiments. As an example, the ecdysone-triggering hormone (ETH) and its receptor (ETHr) are molecules involved in a complex hormonal cascade that regulates molting and metamorphosis (Figure 1C). Since these processes are exclusive to arthropods, homologous proteins are not found in other organisms, making genes from these metabolic and signaling pathways suitable for RNAi molecule development [53]. Several studies have shown abnormal development in species in which *ETH* or *ETHr* genes were knocked down by RNAi [54,55,56,57,58]. Furthermore, our group has previously demonstrated that knockdown of ETHr in CBW larvae leads to lethality and uncompleted metamorphosis [59].

This study generated engineered cotton lines expressing dsRNA molecules for three target genes (*AgCHS2*, *AgVg*, and *AgETHr*) to control the CBW. Furthermore, using a combined approach to target more than one gene simultaneously was an attempt to increase lethality by targeting different processes of the CBW’s physiology (Figure 1). Finally, to increase the stability of dsRNA molecules and avoid the processing of long dsRNA molecules within plant cells, stabilized dsRNA molecules based on the architecture of viroid genomes were used to form a structured dsRNA molecule (dsRNAst). Our findings demonstrate the efficacy of RNAi-based transgenic cotton plants for CBW management. In addition, significant silencing of two target genes in individuals fed transgenic cotton lines was achieved.

## 2. Results

### 2.1. Generation of GM Cotton Lines

A total of 2200 cotton embryos from 4 independent experiments were transformed. After 4 weeks, 150 plants were acclimatized in a controlled room until plants reached the appropriate height to be transplanted. Experimental readouts are summarized in Table 1.

**Figure 2:**
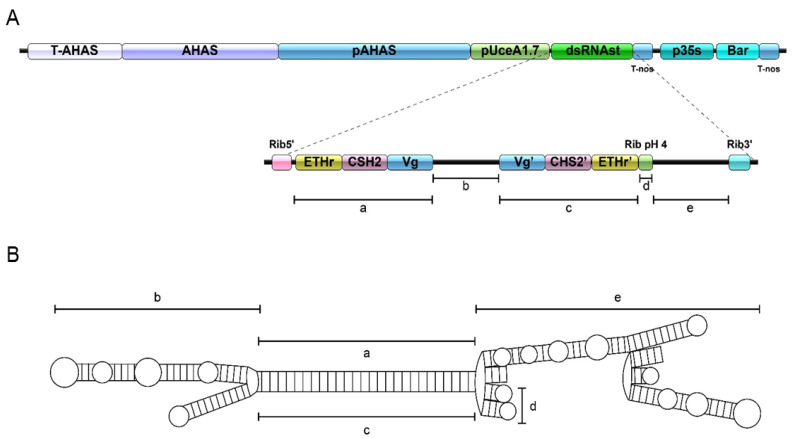
Map of the vector used for cotton transformation. (**A**) Schematic representation of the T-DNA construct for structured dsRNA (dsRNAst) expression in transgenic cotton. The binary vector pCambia3300 was used as the backbone. Imazapyr resistance is conferred to transformed plant cells by the *Arabidopsis ahas* mutant gene under the control of the *ahas* promoter (*pAHAS*), followed by the *ahas* terminator (*T-AHAS*). At the other end of the vector, the *bar* gene is under the control of the *pCAMV35S* promoter and the *t*-*Nos* terminator. Expression of dsRNAst is controlled by the constitutive *pUceA1.7* promoter and the *t-Nos* terminator. The transcribed region of the dsRNAst fragment is highlighted in the figure: at each end of the transcribed sequence are two ribozyme sequences, Rib5’ (from *Selaguinella moellendorffii)* and Rib3’ (from *Vitis vinífera*), flanking the 5′ and 3′ ends of the dsRNA sequences, respectively. The sense (a) and antisense (c) sequence regions contain the target sequences, which *in tandem* contain fragments of sense and antisense cDNA sequences of *A. grandis* genes: ETHr: ecdysis triggering hormone receptor. CHS2: chitin synthase 2. Vg: vitellogenin. The regions encoding incompatibility handles are illustrated in the loop fragment (b) and in the terminal region (e). The (d) site encodes a ribozyme that self-cleaves at acidic pH < 5.0. (**B**) Predicted structure of the mature dsRNAst. Regions indicated by lowercase letters follow those regions described in Figure 2A.

Overall, regeneration ranged from 1.82–10.0% in each transformation experiment, reaching an average of 6.82% (±3.67). From the recovered plants in each transformation experiment, transformation efficiency averaged 12.07% (±1.71), ranging from 10 to 14%. Transformed cotton events were previously screened by PCR of the region encoding the sequences targeting sense and antisense strands of target genes (dsRNA-EF region) (Figure 2). All T_0_ plants were fertile and exhibited normal growth and phenotype compared to WT plants (Supplementary Figure S1).

**Figure 3:**
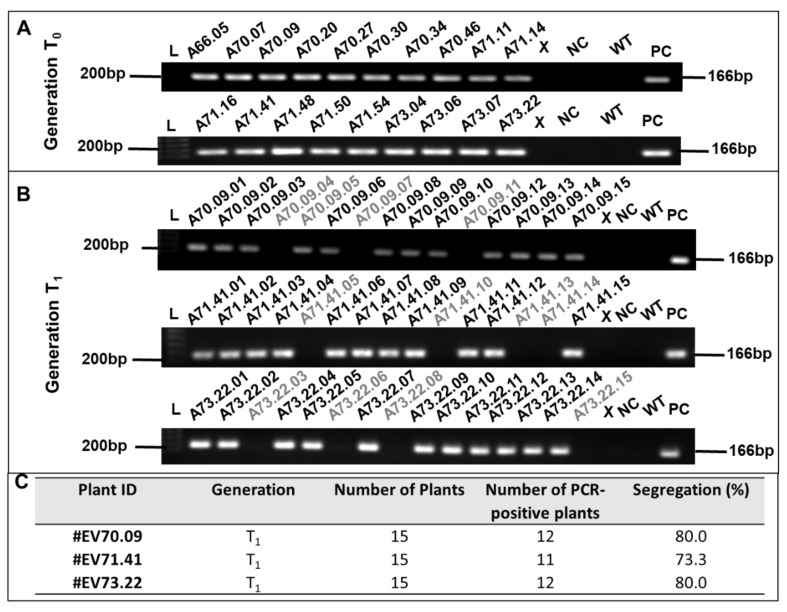
Transgene detection in GM T_0_ and T_1_-recovered cotton events. Putative transgenic plants were accessed by dsRNA-encoding fragment (dsRNA-EF) amplification in a representative sample of putative GM cotton plants by PCR. Amplicon = 407 bp. (**A**) T_0_ progeny selected in Imazapyr-supplemented medium and confirmed by *ahas*-targeting PCR. (**B**) T_1_ progeny selected and confirmed by PCR of dsRNA-EF. (**C**) Heritability among T_1_ progenies. Black letters: positive plants; Gray letters: negative plants; PC: positive control (vector with transformation cassette); WT: wild-type plants; L: 1.0 kb ladder; NC: negative control (ultrapure water). The X represents an empty well.

To determine the heritability of the transgene, the presence of the dsRNA-EF region was confirmed by PCR in plants recovered with T_0_ (Figure 3A). In total, 19 transgenic matrices demonstrated the presence of dsRNA-EF, according to the PCR results. Fifteen seeds of the events A71.09, A71.41, and A73.22, generated by independent transformed events, were sown in the greenhouse, resulting in the T_1_ generation. As described for the parental plants, T_1_ plants were characterized by PCR (Figure 3B). Data confirmed that T_1_ cotton plants also carried the selective marker gene *ahas* and ranged from 10 to 12 out of 15, representing an average heritability of 80% among T_1_ progenies (Figure 3C). Transgenic generations T_2_ and T_3_ of the GM cotton lines also confirmed the presence of dsRNA-EF, based on PCR results (Figure S2).

Based on the initial DNA-concentration and Ct-corresponding values, the following standard curve equations for *ahas* and *UBC1* were obtained: Y_ahas_ = −3.35x + 6.73 and Y*_UBC1_* = −4.5x + 9, respectively. The coefficients of determination (R2) were 0.997 and 0.998 for *ahas* and *UBC1*, respectively, indicating good reproducibility of the linear relationship. The results of the copy number analyses estimate one or two copies in the T_2_ progenies (Table 2). Except for the A71.41.01 event, which had a single copy of the transgene, all events in T_2_ progenies presented two copies of the transgene.

### 2.2. Knockdown of RNAi-Targeted Genes and Effects on Development

**Figure 4:**
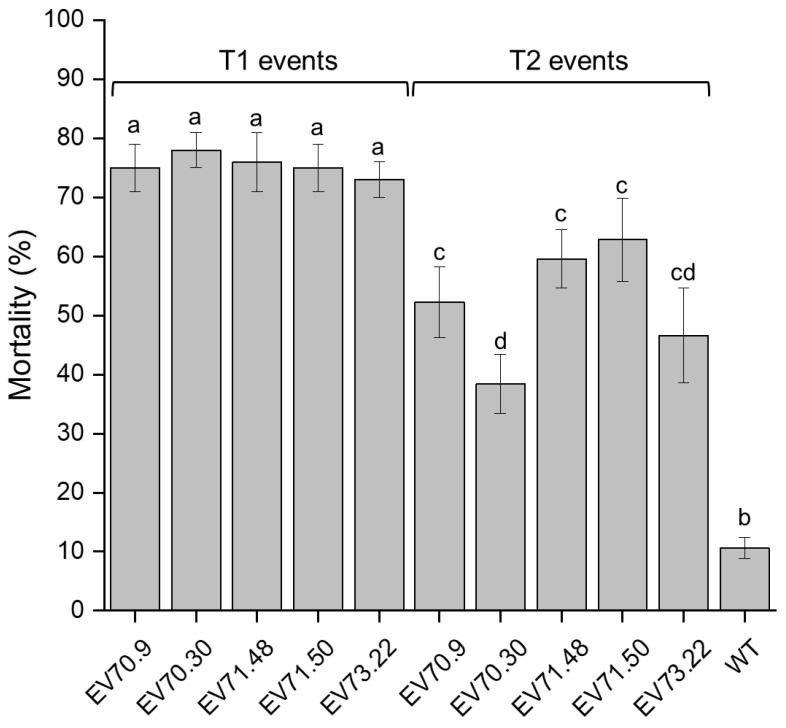
Mortality of *A. grandis* after 30 days of oviposition in cotton floral buds. Individual CBW insects fed on transgenic cotton plants from different transformed events are compared with insects fed on wild-type cotton (WT). Error bars show the standard error mean (SEM) from the six individual plants evaluated per event. Lowercase letters indicate significant differences between treatments (ANOVA one-way-Post hoc Tukey, *p*-value < 0.01; N: 15 insects per plant).

Seven GM cotton events from T_1_ generation resulted in average larval mortality of around 70%, significantly higher than the 10% observed in insects fed with WT cotton (Figure 4). Of these, the following events were assessed in the downstream analyses: 73-22 (Ev1), A71-50 (Ev2), A71-48 (Ev3), and A71-41 (Ev4). Interestingly, larvae fed on T_2_ generation plants exhibited lower mortality and higher variability than their corresponding parents from T_1_ events.

**Figure 5:**
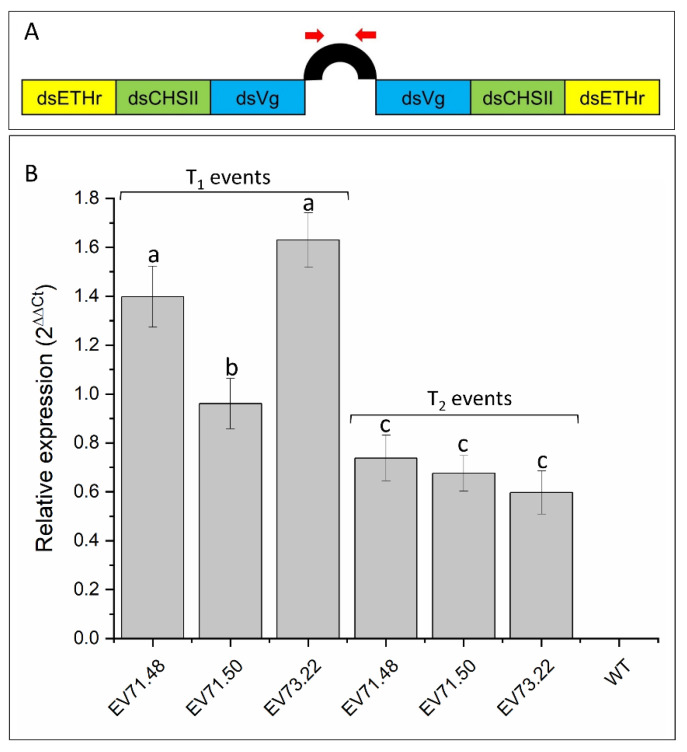
Expression of the dsRNA loop region in cotton buds oviposited by *A. grandis* females. (**A**) Graphical representation of the dsRNA-expressing region in transgenic cotton plants. Red arrows indicate the position of the fragment used for qPCR assessment. ETHr: Ecdysone-triggering hormone receptor. CHSII: chitin synthase II. Vg: vitellogenin. (**B**) Relative expression of transcripts quantified by RT-qPCR using the ΔΔCt method. N: nine buds per plant, three plants per event. Different lowercase letters indicate significant differences between treatments (*t*-test, Bonferroni corrected; *p*-value < 0.05).

Using RT-qPCR, it was demonstrated that the transgenic cotton lines exhibiting the highest resistance against *A. grandis* expressed the dsRNA-EF (Figure 5A) of the transformation vector in oviposited buds. Contrastingly, the expression levels were undetectable in control plants, as expected (Figure 5B,C). Further, expression levels could vary among lines, a fact that could lead to differences in efficacy in controlling *A. grandis*. Additionally, the expression of dsRNA-EF in T_2_ plants decreased compared with their parental events, confirming the same pattern observed in mortality rates.

**Figure 6:**
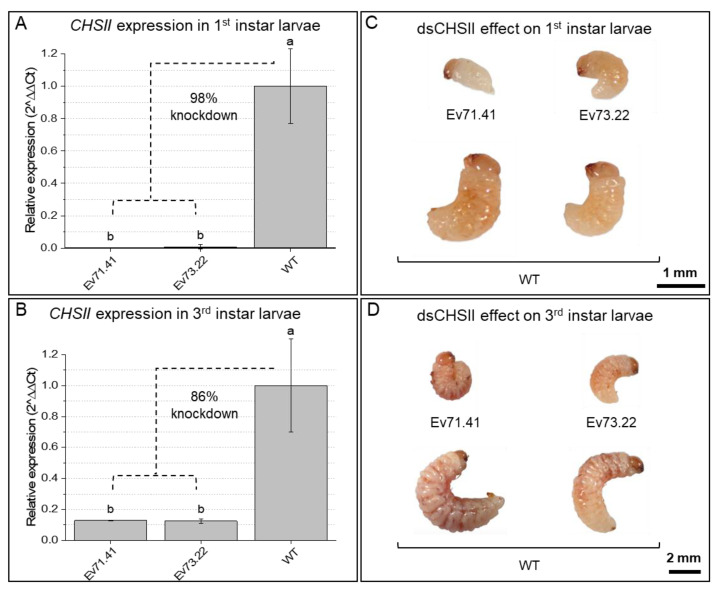
Effects of dsRNA-expressing plants in *A. grandis* individuals fed on cotton after hatching within oviposited buds. Individuals fed on transgenic cotton plants from different events (Ev) are compared to insects fed on wild-type cotton (WT). Different lowercase letters indicate significant differences between treatments (*t*-test, Bonferroni corrected; *p*-value < 0.05). (**A**) Chitin synthase (*CHSII*) relative expression (RT-qPCR) in larvae after 5–7 days of feeding within cotton buds. N: 6 larvae per sample (3 samples per event). (**B**) *CHSII* relative expression (RT-qPCR) in larvae after 15–18 days of feeding within cotton buds. N: 3 larvae per sample (3 samples per event). (**C**) Phenotypic effect of gene silencing in first instar larvae after 5–7 days of feeding within cotton buds. (**D**) Phenotypic effect of gene silencing in third instar larvae after 15–18 days feeding within cotton buds.

After confirming the presence of dsRNAs at the transcript level, the silencing of target genes was evaluated in *A. grandis* individuals fed on buds from four different transgenic lines. With respect to *AgCHS2* expression, larvae fed on buds from transgenic lines showed a significant reduction in expression (from −3.3 to −0.6 Log10 fold) compared with larvae fed on control treatments, especially in the first larval instar (Figure 6A). Accordingly, in larvae that survived after 15–18 days, *AgCHS2* expression was reduced in individuals fed on transgenic events compared with control larvae (Figure 6B). Additionally, it is important to note that the differences in expression were smaller and less consistent (from −0.5 to −0.02 Log10 fold) than those observed in first instar larvae. Alternatively, in insects that completed their life cycle feeding on our transgenic cotton lines, no evidence of *CHS2* knockdown was found in the adult stage (Figure S3). Interestingly, approximately 70% of the first instar larvae collected from buds of dsRNA-expressing plants showed signs of nutritional deficiency, with reduced sizes compared with larvae fed on WT cotton (Figure 6C). The average weight of treated larvae was lower in individuals fed on transgenic lines (approx. 0.7–0.8 mg) than those fed on controls (1.1–1.2 mg) (Figure S4). Further, after 15–18 days of oviposition, insects collected from control buds presented a proportion of first and third instar larvae of 1:10. Remarkably, the proportion of larvae collected from the transgenic lines was 7:3 (first instar: third instar), thus indicating an apparent delay in insect development (Figure 6D).

**Figure 7:**
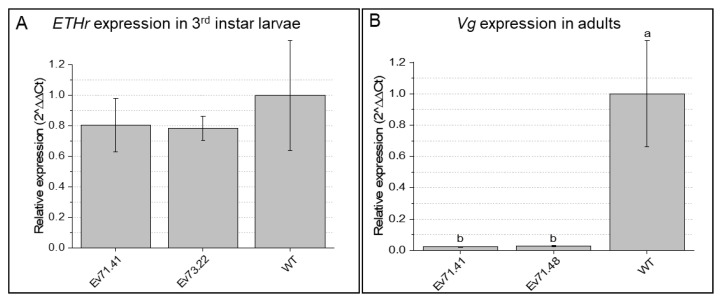
Relative expression of ecdysis-triggering hormone receptor (*ETHr*) and vitellogenin (*Vg*) genes mediated by RNAi in *A. grandis* individuals fed on dsRNA-expressing cotton. Individuals fed on transgenic cotton plants from different events (Ev) are compared with insects fed on wild-type cotton (WT). (**A**) Relative expression of *ethr* in third instar larvae after 15–18 days of feeding within cotton buds. (**B**) Relative expression of *Vg* in adult females after 30 days of feeding within cotton buds. N: 3 individuals per sample (3 samples per event). Different lowercase letters indicate significant differences between treatments (*t*-test, Bonferroni corrected; *p*-value < 0.05).

On the other hand, *AgETHr* expression was not detected in early larval instars or adults. Only third instar larvae showed detectable expression levels; however, no significant differences were found between insects fed on transgenic lines and those fed on control plants (Figure 7A). Moreover, no signals of malformation or developmental abnormalities associated with the knockdown of *ETHr* were observed in any of the evaluated insects. Therefore, the reduction in *ETHr* expression was not proven in our experiments. Finally, the expression of *AgVg* gene, which is only expressed in mature females, was significantly lower in females emerging from buds of transgenic lines and those from WT plants (from −0.1.5 to −0.06 Log10 fold) (Figure 7B).

## 3. Discussion

This study demonstrated that it is possible to disrupt the development and reproduction of *A. grandis* by expressing dsRNAs for multiple target genes in a GM cotton plant. Successful attempts to control coleopteran insect pests through RNAi-based transgenic plants have effectively decreased insect survival when feeding on maize. However, these studies were conducted on species highly responsive to RNAi, such as *Diabrotica virgifera* *virgifera* and *Tribolium castaneum* [12,13,14]. On the other hand, previous evaluations of dsRNA delivery by feeding on the CBW resulted in inadequate RNAi response due to the degradation of dsRNA molecules by intestinal nucleases [29]. Interestingly, continuous dsRNA exposure in species considered refractory to ingested dsRNAs could trigger gene knockdown in other studies [15,60]. Typically, this is achieved by drastically increasing the amount of dsRNA used [49,61], delivering dsRNA by using bacteria [62,63], or through engineered plants that consistently express dsRNA along the time the insect is feeding [64,65,66].

Events of transgenic cotton using RNA interference for pest management are mostly limited to control bollworms (*Helicoverpa* spp.). Efficient silencing of genes related to development resulted in growth inhibition and significant lethality (50–90%) in cotton bollworms fed on leaves of GM cotton [67,68]. Moreover, knockdown of a member of the cytochrome P450 protein family increased cotton resistance to *Helicoverpa armigera* in the early stages of feeding [69]. In our study, we observed variable values of CBW’s mortality in the different GM cotton lines, a feature that could be directly related to the variable expression levels of dsRNA fragments observed among different events. Indeed, increasing GM plants’ efficiency is a critical step toward generating resistant cultivars that would be commercially reliable. Higher expression levels of dsRNA molecules in plant tissues are usually a satisfactory alternative; however, other strategies combining different technologies have been proposed to avoid the emergence of insect resistance in the field. For instance, GM cotton expressing dsRNA and Cry toxins from *Bacillus thurigensis* (*Bt*) dramatically improves the performance of cotton against *H. armigera* [13]. Given the previous evidence of efficient control of the CBW by *Bt* cotton [4], a similar strategy could improve GM cotton’s resistance to this pest.

Herein, we have used sequences of terminal domains present in the structure of viroid genomes, which protect the viroid molecule against the action of the dsRNA processing enzyme complex. In this strategy, the stability of dsRNA can also make it inaccessible to the silencing machinery in insect cells, so we included in the design of the molecule a synthetic ribozyme selected to self-cleave in pH range 4–5 [34]. The pH 4–5 environment can be found in lysosomes after dsRNA molecules are captured and phagocytosed [70,71]. Another approach is for molecules to be protected within organelles where the dsRNA processing machinery does not exist. Therefore, we used in the strategy design the viroid-like structure that chloroplasts use in their replication cycle, thus favoring the availability of dsRNA for the insect [33]. In a transgenic strategy aiming at the expression of dsRNA molecules to trigger the RNAi in the target herbivore insect, it is sought that the dsRNA molecules expressed in the plant system have the following characteristics: long dsRNAs resistant to processing by the gene silencing machinery of plants, in order to create molecules with greater RNAi bioavailability for the target insect [30,72]. In coleopterans, dsRNA molecules larger than 70 bp are essential to trigger the action of RNAi [73,74]. To endow dsRNA with these characteristics, we turned to nature to find the design template for a dsRNA molecule. Viroids are infectious agents formed by a single-stranded RNA molecule that complements itself, exhibiting more than 70% self-pairing, forming a circular dsRNA structure, and not encoding a protein. It is able to replicate and move in plant tissues through plasmodesmata and resist the action of enzymes that process dsRNA [32,74,75].

Our dsRNA delivery system was designed to silence three essential genes, each of which fulfills a different physiological role in the CBW’s development. We reduced the expression of *AgCHS2* and *AgVg*, while *AgETHr* was not consistently silenced. Overall, we can conclude that the deleterious impact on CBW’s survival was mainly due to *AgCHS2* knockdown, as suggested by the phenotype observed in larvae fed on GM cotton. Most larvae could not complete the first ecdysis, with clear signs of nutritional deficiency. Since CHS2 is essential for the maintenance of the PM, its disruption prevents nutrient uptake by gut cells, even in normally feeding insects. Thus, the energy required for growth is not obtained, and the molting process is delayed and even interrupted, as observed in several studies [38,43,45,76,77]. Otherwise, normal *CHS2* expression was found in surviving adults from GM cotton. Since *CHS2* RNAi in adults of different insect species has been reported as lethal [44,78,79], either the treated CBW in our experiment recovered from gene silencing after the non-feeding pupal stage or a compensatory system such as splicing variants could have mitigated the RNAi effect by expressing stage-specific variants of the enzyme. The first explanation seems unlikely since we detected silencing of *Vg* in adults. Therefore, a better knowledge of the CBW’s genome may help elucidate in future studies whether there are isoforms of *CHS2* that overexpressed in adults and were not silenced by our dsRNA sequence.

Additionally, substantial underexpression of the *Vg* gene was observed in adult insects hatching from oviposited buds of GM cotton compared to WT cotton. Previous studies have shown that *VG* silencing in insects impairs egg hatching and reduces female fertility [13,37,48,52]. Thus, we hypothesize that the GM cotton lines may exhibit increased resistance to CBW in future generations of the population that initially fed on buds of the transgenic lines. This is a consequence of the parental RNAi effect on the *Vg* gene.

As for *ETHr* expression, no silencing effects were observed in larvae fed GM cotton. Moreover, the absence of pupal malformations or abnormal molting confirms that the adverse effects typically associated with *ETHr* silencing were not detected in our experiments [57,80]. In some cases, when using dsRNA molecules that target multiple genes simultaneously, knockdown efficiency is decreased for each individual gene as RNAi machinery becomes saturated [81]. However, in our case, we identified a potent silencing of *CHS2* and *Vg* genes, and since our dsRNA structure was designed to be a single molecule, a selective saturation of the RNAi machinery against a specific portion of the molecule is unlikely. Thus, a more feasible explanation for the lack of knockdown of *ETHr* is the spatial expression pattern of the target genes. Differently from *CHS2* and *Vg* that exhibit high expression levels in tissues across the entire digestive system [44,45,77,82,83,84], *ETHr* is not significantly expressed in the gut. Indeed, most of its production occurs in the trachea and epidermis [57,59]. As the uptake of dsRNA molecules in our bioassay occurs by feeding, it is expected that most dsRNAs are absorbed in gut cells and only a minority spread through the hemolymph to different tissues. In insects, local uptake of dsRNA in specific tissues is more efficient and frequent than the systemic spread of the RNAi signal [10]. Therefore, this could explain the absence of *ETHr* knockdown compared with the other target genes in our study. Still, a better understanding of uptake and systemic spread of dsRNA molecules in insects is needed to elucidate issues such as this when targeting more than one gene with the same dsRNA molecule.

The use of multiple target genes to increase the lethality of dsRNA constructs against insects has been implemented with less frequency than the traditional single-target gene design [14,85,86,87]. Interestingly, this approach has been proven effective for insects generally resistant to RNAi. Furthermore, in some cases, simultaneous delivery of two or more dsRNA fragments is performed to mitigate compensatory effects caused by variants in the same gene [88,89,90]. Notwithstanding, our understanding of how insects respond to multi-target dsRNAs is still insufficient. For example, saturation of the RNAi machinery by an excess of dsRNA molecules is well documented and notably influences RNAi efficiency [81]. In consequence, the amount of dsRNA molecules and the number of target genes must be optimized to achieve consistent silencing of all genes in the construct and highly resistant plants.

Furthermore, gene interaction must be considered when selecting target genes. For instance, in our case, it is known that food deprivation affects Vg production and is stimulated by the juvenile hormone and 20-hydroxyecdysone, whose mechanism of action depends on ETHr and has a direct effect on oocyte growth and reproduction [91]. Thus, changes in the expression of one gene could have implications for the expression of the other, making necessary a better comprehension of physiological dynamics between target genes [92,93].

Last but not least, our observations included a consistent decrease in larval mortality and dsRNA expression in plants of the T_2_ generation, which could be related to RNA-dependent DNA methylation, phenomena described for some promoters in transgenes of GM plants [94] and frequently associated to low expression of hairpin RNAi transgenes [95,96]. However, it remains to be clarified whether the decrease in dsRNA amounts observed in our study is derived from epigenetic regulation or if the dsRNA stability (post-transcription) is being reduced progressively in each plant generation, caused by physiological reasons.

## 4. Material and Methods

### 4.1. Construction of Transgenic Cotton

The expression cassette for cotton genetic transformation (Figure 2) was chemically synthesized by Epoch Life Science Inc. (Missouri City, TX, USA) and subcloned into a binary transformation vector. This vector was transformed into *Agrobacterium tumefaciens*-GV3101 and used for cotton embryonic axes transformation, as described by Ribeiro et al., 2021 [4]. The main element of the expression cassette comprises a selection marker gene (*ahas*), a mutant-variant of the homonymous gene from *Arabidopsis thaliana*, which confers tolerance to the herbicide Imazapyr [4,5,97]. In addition, to facilitate the selection of transgenic plants in the greenhouse, the marker gene *bar*, which confers tolerance to ammonium–glufosinate-based herbicides, was also added to the transformation cassette. The dsRNA-EF region of the vector is regulated by the cotton ubiquitination-related promoter (*pUceA 1.7*) [98]. The dsRNA-EF contains sequences targeting sense and antisense strands of *AgCHS2*, *AgVg*, and *AgETHr* (Dataset S1). The full-length CDS (gene coding sequences) of each gene was obtained from the transcriptome of *A. grandis* [24].

### 4.2. In Vitro Cotton Regeneration and Plant Acclimatization

The embryos of the cotton variety BRS372 (Embrapa, Brasilia, DF, Brazil) were transformed according to the protocol described by Ribeiro et al., 2021 [4]. First, plant regeneration was carried out in a growth chamber at 25 °C and 16 h light photoperiod. After 30 days, undeveloped plants with necrotic roots were removed. Seedlings longer than 2–6 cm were transferred to 0.7 L pots with soil/substrate (50:50), covered with plastic bags to maintain humidity, and placed in an acclimatization room at 25 °C and with a 16 h light photoperiod. Plastic bags were removed after one week. Well-developed plants were characterized by PCR (polymerase chain reaction), and putative transgenic plants were transferred to 5 L pots containing fertilized soil and Vivatto commercial substrate (Technes, São Paulo, SP, Brazil) (3:1). Then, plants were placed in a greenhouse under 70% humidity, 25–35 °C, and 12 h of light to obtain the transgenic segregating seeds. The budding flowers were sealed to avoid cross-fertilization. Thereafter, 15 seeds from each T_0_ individual were planted and acclimated in the greenhouse, thus originating the T_1_ generation. Transgenic events of the T_1_ generation confirmed by PCR were bioassayed with the CBW.

### 4.3. DNA Extraction and PCR

Genomic DNA from putative transgenic plants was extracted from the upper young leaves using the DNeasy Plant Maxi Kit (Qiagen, Hilden, Germany), according to the manufacturer’s instructions. DNA concentration was spectrophotometrically determined (NanoDrop 2000, Thermo Fisher Scientific, Waltham, MA, USA), and its integrity was assessed by electrophoresis in a 1% (*w/v*) agarose gel. To confirm the presence of the dsRNA-EF, the Extract-N-Amp Plant PCR Kit mixture (Qiagen, Hilden, Germany) was used from 40 ng of genomic DNA and specific primers (10 mM) (Table S1) for gene amplification, as follows: 95 °C for 15 min; 35 cycles (95 °C 45 s, 60 °C 45 s, 72 °C 1 min) and 72 °C for 10 min. The genomic DNA from the wild-type plants was used as a negative control, and 50 ng of the binary vector was used as a positive control in the PCR reactions.

### 4.4. Transgene Copy Number Estimation

To estimate the transgene copy number, a total amount of 40 ng of genomic DNA from wild-type (WT) and genetically modified (GM) cotton events was used as a template for the qPCR analysis. The *ahas* transgene and the endogenous ubiquitin 1 (*GhUBC1*) of *G. hirsutum* were cloned in the same plasmid *pBSK*::*ahas*::*GhUBC*, which was used for performing the standard curve, as described by Yi and Hong, 2019 [99]. The absolute quantification of the *UBC1* and *ahas* genes was performed according to Ribeiro et al., 2017 and Paes-de-Melo et al., 2020 [4,100], using specific primers (Table S1). For this purpose, the *ahas* marker gene was selected as the transgene (target gene) and *UBC1* as the endogenous normalizer control. The amplification curves for *ahas* and *UBC1* genes demonstrated good reproducibility over their entire linear coefficient. The determination coefficients (R2) were 0.997 and 0.998 for *ahas* and *UBC1*, respectively.

### 4.5. Evaluation of the CBW’s Survival in Transgenic Cotton Plants

Transgenic lines and WT cotton plants were challenged with fertilized CBW females in greenhouse bioassays. For this purpose, T_1_-transgenic and WT cotton plants were grown in the greenhouse until the reproductive stage. When the first floral buds emerged, the fertilized CBW females were released into the greenhouse. The CBW individuals used in the bioassays were obtained from a population reared in WT cotton in a greenhouse at Embrapa Rice and Beans (Santo Antônio de Goiás, GO, Brazil).

Individual buds of each plant were examined every 48 h. Oviposited buds were isolated within organza bags, and the date was recorded for each bud. Aborted buds were collected and maintained in a room with controlled conditions (25 °C and 12 h light photoperiod). After 30 days of oviposition, the buds were collected and opened with a scalpel. Emerged adults were recorded as survivors, while malformed individuals or intact buds were registered as deaths.

### 4.6. Expression Analysis of dsRNA Molecules

In order to confirm the expression of the dsRNA sequences in the buds of transgenic cotton lines, a fragment corresponding to the loop region of the dsRNA structure (Figure 2) was amplified by RT-qPCR using specific primers (Table S1). Three different transgenic lines with high resistance against *A. grandis* (>70% mortality) were selected for further analysis. Three WT cotton plants were used as control. Biological replicates comprised a pool of three buds used for RNA extraction. Total RNA was extracted from 100 mg of buds using the InviTrap Spin Plant RNA Mini Kit (Invitek, Berlin, Germany) according to manufacturer’s instructions. Then, DNA was digested from 2 µg of total RNA using 1 µL of DNAse I (1 U/µL) RNase-Free kit (Invitrogen, Waltham, MA, USA), according to the manufacturer’s instructions. Next, the purified RNA was used as a template to synthesize the first strand of cDNA with a MMLV-reverse transcriptase enzyme (Promega, Madison, WI, USA). The synthesis was performed in a 25 µL reaction containing 5 µL of 5× reaction buffer, 1.5 µL of dNTPs (10 µM), 200 U of MMLV-RT, and 1.5 µL of random primers (10 µM) (Invitrogen, Waltham, MA, USA). The temperature for the primer melting step was modified to 85 °C for 5 min. The transcription step was carried out at 37 °C for 60 min. Finally, the qPCR reaction was performed by using GoTaq qPCR Master Mix (Promega, Madison, WI, USA), according to manufacturer’s instructions. The cDNA used as a template was diluted in a 1:20 ratio. The ubiquitin 14 (*GhUBQ14*) and serine/threonine–protein phosphatase PP2A-1 (*GhPP2A1*) genes were used as endogenous normalizer controls in the qPCR analysis. The following thermocycler program was performed on a CFX96Touch Real-Time PCR Detection System (BioRad, Hercules, CA, USA): 1 step (1×): 15 min at 95 °C, 2 steps (40×): 30 seg at 95 °C, 30 seg at 60 °C, and 30 seg at 72 °C. RT-qPCR data were processed with MINER qPCR software (Nanjing, Jiangsu, China) [101] to obtain primers’ efficiency and with qBASE plus (Biogazelle, Grent, Belgium) for expression and statistical analysis. Expression was determined by the 2^−ΔΔCt^ method, and statistical differences between treatments (pairwise) were calculated by *t*-test with Bonferroni correction (*p*-value < 0.05).

### 4.7. Validation of RNAi-Mediated Knockdown

To determine the effect of transgenic cotton lines on the expression of the three genes targeted, different developmental stages of *A. grandis* were chosen for downstream analysis. Bioassays were performed as previously described. First instar larvae were collected from oviposited buds after 5–8 days, third instar larvae after 15–18 days, and adults after 30 days. Adults were separated by sex, and only females were used in the following steps. Then, insect samples were frozen and stored at −80 °C until further processing. For the first instar larvae, six individuals were used in each sample. Otherwise, for females and third instar larvae, three individuals were used for each sample. In total, three independent samples were used for RNA extraction in all cases. Total RNA was extracted using Trizol reagent (Invitrogen, Waltham, MA, USA) and treated with DNAse I (1 U/µL), RNase-Free kit (Invitrogen, Waltham, MA, USA), according to manufacturer’s instructions. First strand cDNA was synthesized from 2 µg of purified RNA using OligoDt30 primers and MMLV-reverse transcriptase enzyme (Promega, Madison, WI, USA), according to the manufacturer’s instructions. Conditions for quantitative PCR and expression analysis were the same as described in the previous topic. The reference genes used encode two ribosomal proteins (*RPS26* and *RPS11*). Primers used for these analyses are listed in Table S1.

### 4.8. Phenotypic Effects of dsRNA-Expressing Plants on CBW

Insects were collected following the methodology described in Section 4.5. Evaluation of the CBW’s Survival in Transgenic Plants. The weight of collected insects was measured using an analytical balance SECURA5102-10BR (Sartorius Lab Instruments, Otto-Brenner-Strabe, Germany) and photos were captured using a digital camera Leica DFC310 FX coupled onto a stereo microscope M125C (Leica Microsystems, Wetzlar, Germany).

## 5. Conclusions

In summary, our data remark on the previous efficacy of viroid-structured dsRNAs in the gene silencing of refractory insect species and a significant improvement in insect pest control by combining different targets in the same dsRNA-encoding fragment in genetically engineered plants. This represents an important step for plant biotechnology and pest management in Brazil as an alternative method to current pesticides. It is worth noting that our GM cotton is the first to confer resistance to *A. grandis* using dsRNA molecules, and it came shortly after a previous study with a similar approach, but which used Bt toxins instead of dsRNA [4].

We highlighted that CBW mortality in transgenic cotton expressing dsRNAs for *CHS2*, *Vg*, and *ETHr* was surrounded by 70%, fluctuating with the dsRNA expression in the transgenic plants. Furthermore, insect gene-silencing features highly depend on each target gene’s expression pattern and function over the insect lifespan. Data over *CHS2* silencing showed that insect development was severely impaired in transgenic plants, resulting in malformed first and third instars’ larvae. However, no disparity was observed for the attempted silencing of *ETHr*. The *CHS2* knockdown likely impaired nutrient uptake in the insect’s gut, justifying the phenotype and high levels of insect control in the GM cotton. This effect was enhanced by the *Vg* gene silencing, which could affect the proliferation of insects continuously fed on the transgenic plants. Overall, all data underscore the dependence of insect pest knowledge in wide-genomics, transcriptomics, and physiology, as essential genes can change their expression profiles at different life stages and interplay different roles throughout, allowing a better characterization of targets potentially applied in biotechnological crop breeding, especially for insect pest control.

## Figures and Tables

**Table 1:** Cotton plant regeneration and transformation efficiency data from eight independent cotton embryo axis transformation experiments. Transformation efficiency was calculated based on the number of PCR-positive plants divided by the number of transformed plants, which were selected by herbicide (Imazapyr).

#Transformation Experiments ID	Number of Inoculated Embryos	Number of Transformed Plants	Regeneration Efficiency	Number of PCR-Positive T_0_ Plants	Transformation Efficiency (PCR-Positive T_0_)
A66	550	10	1.82	1	10.0
A70	550	50	9.09	7	14.0
A71	550	55	10.00	7	12.7
A73	550	35	6.36	4	11.4
Total	2200	150	6.82 (±3.67)	19	12.07 (±1.71)

**Table 2:** Transgene copy number estimated by qPCR in T_2_ GM cotton plants. Copy number was estimated by qPCR analysis. A standard DNA curve (1–10^−5^ ng) was obtained using a binary plasmid carrying an endogenous reference gene and the transgene against the linear cycle threshold (Ct) values.

Plant ID	2xAHAS/UBC1	Estimated Copy Number
Ev70.09.18.02	1.53	2
Ev70.09.18.05	1.49	2
Ev70.09.18.08	1.52	2
Ev70.09.18.13	1.52	2
Ev70.09.18.17	1.49	2
Ev71.41.01.01	1.36	1
Ev71.41.01.03	1.23	1
Ev71.41.01.08	1.37	1
Ev71.41.01.10	1.40	1
Ev71.41.01.12	0.79	1
Ev71.48.07.01	1.54	2
Ev71.48.07.02	1.64	2
Ev71.48.07.08	1.49	2
Ev71.48.07.11	1.54	2
Ev71.48.07.14	2.11	2
Ev73.22.12.02	1.51	2
Ev73.22.12.05	1.50	2
Ev73.22.12.08	1.54	2
Ev73.22.12.16	1.55	2
Ev73.22.12.19	1.70	2
WT	−0.16	0

## Data Availability

All data supporting the findings of this study are included in the manuscript and supplementary materials published online. Original images from agarose gels are given in a supplementary file (Figures S5–S11).

## Supplementary Materials

The following supporting information can be downloaded at: https://www.mdpi.com/article/10.3390/ijms232213713/s1.
