# Supplementary material for: Stabilized Double-Stranded RNA Strategy Improves Cotton Resistance to CBW (Anthonomus grandis)
Source: Int J Mol Sci. 2022 Nov 8;23(22):13713. doi: 10.3390/ijms232213713 (PMC9691246; doi:10.3390/ijms232213713)
Supplement: Supplementary file 1 [file ijms-23-13713-s001.zip › ijms-1820823-supplementary.pdf]

>Agethr

atcccttcgtagaactgactgtagctcacgcctcggtactgaccatcttagccataagc
ttcgaaaggtactacgccatatgtaagccattaaaaagcgggctatatatgtacaaaaa
actagggcttctctaatttgccttttggcatggtttattgctgcggtgtttaccagccc
gatattaagcatagccgaatacaagctactagacgacgacggaacgctagtaccagcct
gtcatacctcggccaacactatctggtccgccctgtatttttgggctagcattttcct
atttttcwwtagttcctcttgtagtgcttatcgtactttattgcattattgctaaaaat
ttaatatttaacgggactagtttcctagtaataaacatattgatagttattcgatgcgt

>Agchs2

agcctaatccccgaacaaggattttgaaatcatacttggtaatggatattttgttagta
tcaaataaaaattaatcttatcgggaaacacatcacttcgaatgtcgtcggttgatcgt
gtctttctcagttctatgataatatgtttatttttattttaactaacgcacggtaaatc
ggacctacgaaactgaaaagtgcagtcagttaagtgttttgtgacccaatttgtgttct
accgcacaaatggacgaggattttgacgactttgacgattttgacgacgaggattcttt
gttaggatcaacagagaggcagggcgaagaagtgaaaccatggaacaccttcaaggtga
tccacaggaaaacggccagcggatccacagtcgaaaacaaactggtggacaatggagtc
aaatttctcaaaatcgccaccattttatcacattttggtggtactaggtacggcagt
actatccaagggattgattctcttaatgacctcacagatcaagaagaacgtcactagga
attattgcaataaaggactagatatttcaaggcaatacatcttctcagttcccgaagtt
gaaagagccacctggatatggtttttgatgctcgcctactttgttcctgaatttatgac
attctttaggtcaggacgaattctactattcaaaacgagactctatccaacccttacgg
agtttctcagcctattagtaacagaatgtttaccagcaataggcagcagtctgctaatt
ttctgcgttctaccggaactcgatgtggtcaaaggagccatgctcactaacgcaatgtg
tgtgattccagcattaatgttggtttttacgaaaattggtacgaaaaagatctcaatag
tcactgaaattgcggatattttagcattgctagcgcagctatcagcgatgctcgcttgg
ccactaataaatcacgatgaacctatactgtggcttataccaatttgtgccattttaat
ctcgtttggttggtgggagaactttgtttcgttatcatcaccaataaaattggtacgac
aattggccaaatccagaaaagagtttcccacccgcaagtacttttgctactttattatt
tcaccggttaaatgtataatttcttcgcgaccactatagcttgcatatattttaaaga
tggcgatgtgaacttttttattcgagaacttttccgatattttctggaatcatccgatga
ccgttaccgaggttgttccacaagtgacaggtaccaacatcaccgtagatgacgcaatc
agcactgggatagtccacataatctacagcgatatcaatcttcaggcagccatttgggg
cgtcagcgtcgtttccgcttatgcttgctacgcctttggcaagtttgcttgcaaaatta
tgatccagggttcaagttttgcttttccgatcgctttaactgtgcctttcctcatatca
gctctggtgatcttttgtggattctatgccaaagacgtgtgtgccttctatgacttctt
accggcatatctcttctttaactcaccacctttgcttaacctcttgaacttcgtggaga
ctcaacatccgtatttatggctgttttggctgctttcccaaatatggatcaccaggcat
atttggaccaacaataactcgaagctggcttccactgaaaagttgttcatgcgtcccat
gtacgacgggatcctgatcgatcaatccattgcaatgaatcgcagaaatgtcgtggata
aacttgtagaggaggagaaactggcagacggattggaaagcaagaatatcattgacgag
aataaaataacaagaatctacgcttgcgctaccatgtggcacgagactccggaggaaat
gatggagttcttcaagtctattttcaggatggacgaggaccaagcttgctaccggattt
ctaggcagtacttacagtaccccggcgaagggtattatgagtgggaaacacacatcttt
tttgatgatgcctatttgagaaaatcagtaaacgacaacgatccaatgctaaactccta
cgtgaacgattttattgcaaccatacccaaagccgcagaagaagtgcataagacaacag
taaaaattcgacctcctacaatatatcccactccttacgggggcagactaatatgggtc

ctaccaggaagaacaaaactcatcgtacatttaaaagacaaggctaagattagggctaa
aaagagatggtctcaagtgatgtacatgtattacctgttaggacacaagttaatggata
atgaagaaatggatgataaaaaggttaagctaagatcatttaacacgtacatcctggcc
ctcgacggagacatcgatttccaaccggaagcagtcgctttactagtccagtatatgca
aaaaaagagcaacttaggagctgcttgtgggcgtattcatcctgtaggttctggaatta
tggcctggtatcaaacgttcgaatacgcagtgggccattggatgcaaaaagcgaccgaa
cacgtcataggttgcgttctttgttcgccaggttgtttctctctctttagagctagtgc
ccttatggaccacaatgtaatggctagatatacgacgcgttcttccgaggctaggcatt
atgtacaatatgatcagggcgaagatagatggctctgcaccctcctactgcaaaggggt
tacagagtggaatattctgctgcctccgatgcctatactcactgtccagaaggtttcat
cgaattctacaatcaaagaaggagatggggaccgtctaccactgcaaacattttggatc
tgcttgaggatagcgatcacatcaagttggttaacgatgatatatcctctctatatata
ttttatcaagtgattttaatgattggcacagtgattggtcccggaacgatatttctcat
gttggtcggtgcgtttgttacggttttcaatgtttcccagtttacggcgttgtggctca
atgttggtcccattttgtttttcgtgctcacttgtataatatgcaaatcggatacgcag
ttaatggtggcagcaatactgagcgcgatctacggcctagtaatgataatggtgctcat
aggtgtcgcgatgcagatctacgacgatggtgttctggccccttcttctctgttcttct
tcttaatgatgggcgaatacgtggtagctgcaatgctccatccaaaagagttctactgt
cttaaatatggcgccatctacttgatcaccgttccaagcatgtacatgttgctcatcat
ctactctgtgtttaacatgaataacgtcagttggggcaccagggacgtcagcgtagcac
ctccactgccagagggccaaccgaaacctccaccaaaaaaagaaaagtccttcgtagaa
gacgtactcgataaaatgaagaagttctttatggcatgttgctcgggagactccaagca
tttagttatgatcagtaattctttgacgcacattcaatcaaaagtggaacaaattgagc
agaaagtcgaggatttggagcggatcactttggatccagatgcggcagtgccaaggaaa
accatgggaaaaaggaaaaccactattattgaagggtctagggcgtcgagacagtcact
ccggaaatcaacgatgaacaaaccaggggccatcccaaatgaaaacgccaatgcctcta
ttgccgaagatgatgaagaagattatgatgaggaagagtccatcggtagctcagatgat
cttcaaaacaatagctggttttacgcgggtgaactcctaaggggacgagtgactttctt
ggataaaaaggaggaaaagttttggaaggagttgctagatgcttatttgcatcctattg
aagatgataaggaaaaagtggccaaagacttgaaagacctgagagacagaatgaccatg
agcttcttcgcactaaacgtatttttttgtcacggtagtatttcttcttaccattaagaa
agacatacttcacttaaattggccgttcaatcccacggtgaacttcacttacagtgccg
ccagcagcatcaatgagattgtcgttgaaaaaacctacttgcaactggaacctatagga
ttcgtgtttttgatcttcttcttcgccctcatggggggtgcagttctttgggatgttgct
ccatcgatttggcacgttttcacaaattatggccaacaccgatgtcgagtttggagaaa
agaagatagaaaacttgaccgaggacgaactactagaaaaagactctataaaaatcgta
aaaaaactggtgaaactcaaaggcattaacggtgacgacgagaaagacgaggaagtaga
cgctcacgtatccagaaggaaaaccgtggcggacttggcgaaaaacaaagacagaaaac
gtgcagtcatcaacgacttggattccgcctttaaagcccgtatggcaaaaatacgtaaa
ggagaagacgctggagtggctttacctaggaaaactttggcagcaattcacagaagaag
aacaacagttcttaagagaaaatcaatgatgccgcagttcaatcccagcgcgtttaagg
cgtttaatcagcattatggagaggagagtcctagcaggactatacatcatatagagaat
cccgggtatgatgatgatcctgctgacgcgtagattttatgtgctagttgttttagaat
ttttaataaataattttgggtcttaatatatttttttttaattatctcatacagaaattc
ctaaactcaatctaagaggtgtcttcttttctgtttttttcttcttctgagatcg

>*Agvg*

atgtggtcaacagtggctttgtgtttgttggttggactttcttatgtctcctcatcctc
gccagcctggaaagataatacggagtatgtctattcagtaaatggtcgtacactaaccg
gacttgaagaaaccgctgatcagtattctggtgtctttctagaagccaaattacatctc
tctattcgtcctgatggaaagctccaaggccgaatttctgaacctaaatttgctcagat
tctctctcagttacctgatgggtggaaatcagagattcctgattcacaaattagttaca
agcaattacaactttctcaaaaaccatttcaactggttttagagaacggtttaattaag
agactgattgtggagaaggacactctaaactgggaggccaatatcatcaaaagtattgt
aagtcaattccaaatggatttacagggagaaaatgccttgcagaaccccaccagcagct
ttcccactaatgaatatatggatgccgtttttaaaaccatggaagaaactgtaacaggc
aaaactgaaacgatctatgacattcaccgtcttccagaatatttagttcaaagtcaacc
ttggattgctcctcaatataaactgaaaggtgaaggagaccttattgaagtcataaaat
caaagaattataccaacgctcgtgataggccttcgtaccactatggctttggagaaatt
gaagaatcagaaccaactgctaataaaatgggacagttctttataagacaatcaaattc
tagagctattcttactggaaaaccctctagatatattattcaaagcacttataccgtta
acaagatcatggttaatccaattcttaagaataaagaaatgggatctattacaagtatg
gtcaatgtcacattactagaaattaacaaccaacaacaacagcctgaagaactttccaa
tcctttagacattggaaatttagtatacacttatggacaacctaaaaacaaccaagtac
attcaaaattaaatgaaaatctcatggaagattcatccagcgaagagagtagtgaacag
gaaatgactcatagaagattccgccggtcagccaactctcttacaaaacaatggagaga
aagttctgaagaatggaatcaacaacaacaacaaccccgtcctcagttaactagagctc
cgcattccccacttcttccatcaatggttggctatcacggaaaatcaattaaagaaaat
aaagactttgatatcagacaaaatgttgaaaatctagttacggaaatcagtgacgaaat
taagcaatcagaaaaaaccatctctaaacataccttggataaatacactatcctgaata
ctcttgttcgcctgatggacgaagacgatattcaatttgtagccgagcagatgtattct
cagatgaaaaacggacagcagcgttatacctggagcattttccgtgactccgtagctga
agctggaactggaccagcccttttgaacattaagaaatggatagaaaccaaaaaaatcc
aaaaaactgaagctgcacaagtaattggtactctagcccaatcaacacgtttcccaact
gaagaatatatgcgcaagttctttgaattggcaactgaaactcaagtcagacaacaaga
aacactgaatcaaacatgtattctatcttataccaacttagttcacaaagtatacatca
atagaaatgaatctcacaatcagtttccggttcatgcctttggaagtttctatactaaa
aaaggaagagaatttgtcaagactactgtaatcccccatctcaagcaggaattagaaaa
agcgattagcaatgctgataacaataaaattcatgtaatgatccgagctcttggaaaca
ttgggcacaaatctatcctaaacgtgttccaaccctatttcgaaggagaaaagcaagtt
tctcaatttcagagattaatgatggttgcctgtatggaccgactggctgattgttatcc
gcatattgcacgttctgtttttataagatctaccaaaatactgccgagcttcctgaaa
ttcgtgttgtagcagttcatcaacttatcagagctaatccacccgtagagatgcttcaa
cgcatggctcaatataccaacactgactcccaagaagaggttaacgctgcagttaaatc
tgtcatcgaaagctcatgtaaacttgaatcatccaaacatgctgaattacggaaagcag
ctcagtccgctagacctttgctcacgaagaagcaatatggaatggaacaaagttatatt
aacttgcgtgattatgtggctgaacaaatgggtcttgaacttcacgtgcaaagaactag
ccatagtagtgctgaaagcagtttcccgaagattatgaaattccaactccaccaacata
accatggaatgaaacagcacattctgtctaccgggggaatgatatccagcatcagggag
cttttgaatgttttatatagacaaactgaagtcttccaacaagaaaaatctcagagatc
gcaggaacaaggtaaagacaacgaatggtccagcgccaatattgctcggttaatgaact
atgaacgcgatgaacgtgaacagttagaagctattatttatgctcaagttgaagatgta
caaaaactttggtctttcgataaccaaactttagagcatctcccagaagttattcgtca

gcaagaagaaatttacagacaaggaaaagattttagctatgtcaagcttaaacagctaa
atgaaatggcactctccttccctactgaaatgggtctgcctttcttgtatacctatgat
gtgcctgtgttgatgaaggttgaaggaaaaattagagctcttgccaaccctgctatttc
cagaaacaacaaactcactaaacctgaacaaatttctactgaaattaaagctagagtta
cctgtactggcaaaactcaaagccatttgtctttcgtaactccatttgatcatcaaatc
tatatggctggttatgacaagaatatgtatgttagtatcccagtcaacgcaaggctcga
aatggatgtgaaatctaaggaagctaaaattgaattcgaagttgaacaacagcaacaag
attctcgtttagtgcacattactagcactccctacacctcaagaagtgatgttatggca
attagtcctgtagctttgagaccaaacacatatgtaattaagtcccacaggaacaacca
cagatattttgacttcaatttcggcaaaaaagaaactggtttaacattccggggatggg
gacaccatcctgaacaaagtatcggatttaatgatttagtatctatgtggcaatcacgc
ggagttgctggtgtatgggaacaattatgggacaaatgttctactgaatatagcgaagc
taccatcagttttattccaagtcaatctactacccgtaaagctactttccgtattaatg
ttgatcaaaaataccagaaacaacctgagactcaaagtccagaagatcttctaacctta
aatcaactttcatctaaattgcaaaaagatgaacctaaacaacgtcaacaagaaattaa
aaaacacgtcgggtccggtatcaacagcgctttattgtcatgctccgacatctcgcttg
aatttgagggagataagaaatacgaacacgtagtcggttttgccgttgcaaagagtaat
gctgatcctaaatcaagagttatgttctattacaaaaacaaaaatgaaaataaacaggg
agctttggaaatcagaagcgaaattcctaacaccaatggattgaatctagatgattcct
tggatactgagccttccaccaaatacaacatgcgattgcagtacggaaatagcgaaaat
gatgcttttgaaatttctgctcaagctcaacttagtagaagccaggaacgaaagcaata
tttaattaaccaagatccattgtaccatgtatgtaaggagcaaatgcaacaaaagaact
tccaactaccagcttgccaaaacatgactattaaagccaacttcctcgaccacattaaa
tatcaagttcagtatcaaaaattgaactggaagctggttgaaactttggaaggaatgtt
taagggattgagagtattatactatccaatgactgaaattaaatcaatttcttctgtcg
gtcaaaatgtcgtagaaggagaagttcaattccagccagaagatttagacaagtcaat
gtaacagtaagaaatactgatgaagaaactgtattcttcaacatttctttaaataatga
acttttgagaacgctcctggtccctcatccagtgttccatgctaaatgtagatttgctg
gtttaatgcaaggtcaacaaaattacagacccacatgcgtgattgaccaaaccactgct
caaacgttcagcaataagacctactcagttaatcttgacaaagaacccactgtagtaat
gcagtatgttcccaaagatgctagagttaatggacagcagtcaaaatccgttgaacaat
tactgagagaaagcattgaaaactatgttgttcttgtccgtcaagtcgctgccaatcaa
aaagaagtaatcatcaacttaaaccatcccagaactcaaggaaaaactgttaagattga
aatgaaaccatcagaagacagacaaaaatctgctcgcaacccagctgctaaagtcacta
ttgatggacaagagatgcacttcgatgataagcaaatcgccgataagtgtgatggatat
gttcaagtatatgctttacccaacggagaagttaaacttgaagtggaagatgcttttta
cctgatttatgatgggcaacgtgtcaaagtaactgccactggcaacaagcttcgagact
cagtatatggtttgtgtggaagattcagtcaagataaacatgaagactttacagttcct
tcaaactgtgttacaagagatactcgtaagtttgtggaaagttaccaagttgaaaaggg
tcaacaatggcgcaactctccttctgaacaatgcataaagaaagtattaccattgtaca
ctaatgtaatctccaaccagaatggttcccaaatgagaacaaaacttgccagtggaact
gtaatgaaacaccgctacattgaagaaaatggtgaaatttgctttaccattcgcccact
accagtctgcaacacctcagtcaaacaagtcgtgacgaaaaacgttcctgttcactgta
tccaaggaaccaagaccgcttattactacaaatccctaattgatcaaggaggaaaccca
gatttcagccgtaagagtgaaactaggacagctcgcatggaagtagcagcacaatgcaa
ctaa

>dsAgethr

tcgggttgtcccagcccgatattaagcatagccgaatacaagctactagacgacgacgg
aacgctagtaccagcctgtcatacctcggccaacactatctggtccgccct

>dsAgchs2

aagtagacgctcacgtatccagaaggaaaaccgtggcggacttggcgaaaaacaaagac
agaaaacgtgcagtcatcaacgacttggattccgcctttta

>dsAgvg

tagccgagcagatgtattctcagatgaaaaacggacagcagcgttatacctggagcatt
ttccgtgactccgtagctgaagctggaactggaccagccct

Supplementary Dataset S1. DNA sequences of target genes in CBW used to construct the cotton transformation vector. Italicized headers indicate the complete transcript sequence obtained from the CBW's transcriptome. Headers in bold letters indicate sense strand sequences of DNA encoding the dsRNA molecules.

Gene	Primers (5' – 3')	Organism	Used for	Amplicon length (bp)	Efficiency (Miner)
Target1	F- gactacacccggctgatgagt	*G. hirsutum*	PCR	407	--
	R- atccgccgatccgtacttac				
Target2	F- gttctgccatggggtagagct	*G. hirsutum*	qPCR	106	1.85
	R- atccgccgatccgtacttac				
UBQ14	F- caacgctccatcttgtcctt	*G. hirsutum*	qPCR	75	1.82
	R- tgatcgtctttcccgtaagc				
PP2A1	F- gatccttgtggaggagtgga	*G. hirsutum*	qPCR	100	1.86
	R- gcgaaacagttcgacgagat				
CHS2	F- aaggcattaacggtgacgac	*A. grandis*	qPCR	121	1.91
	R-tccaagtcgttgatgactgc				
ETHR	F- ggtttattgctgcggtgtttac	*A. grandis*	qPCR	110	1.91
	R-gatagtgttggccgaggtatg				
Vg	F- tcatcaaatctatatggctggttatgac	*A. grandis*	qPCR	201	1.88
	R-gctacaggactaattgccataacatcac				
RPS11	F- acccacggttttccttaacc	*A. grandis*	qPCR	123	1.89
	R-gacgactccggtgaggatac				
RPS26	F- tcccaaaagacaaggcaatc	*A. grandis*	qPCR	150	1.96
	R-ccttcttgctcctgttacgc				
AHAS	F-gtcactgggttaatatctctcgaatcttgca	*G. hirsutum*	qPCR	257	1.99
	R-cctacttccaatgtctgattagtgcttctgg				
UBC1	F-tggcattatattgtcattgttactatcc	*G. hirsutum*	qPCR	121	1.99
	R-accatgttatcttattctaagacaagctc				

Supplementary Table S1. List of primers used in PCR and qPCR experiments. Target 1: fragment for PCR confirmation in the transformation vector. Target 2: loop region of the dsRNA-EF fragment. *UBQ14*: ubiquitin-14. *PP2A1*: serine/threonine-protein phosphatase PP2A-1. *CHS2*: chitin synthase 2. *ETHr*: ecdysis-triggering hormone receptor. *Vg*: vitellogenin. *RPS11*: ribosomal protein 11. *RPS26*: ribosomal protein 26. F: forward primer. R: reverse primer.

A

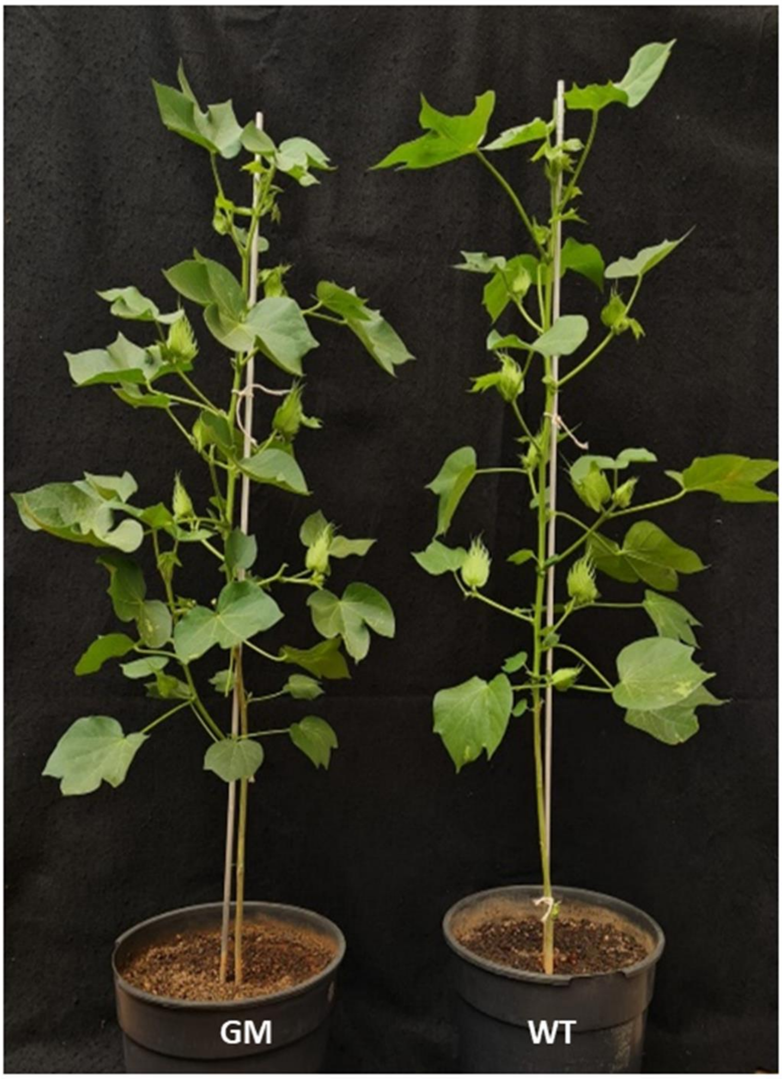

B

Generation		# flower buds per plant	# seeds/cotton boll	Weight of fiber (g)
T0	GM	22.6 (± 1.42)	26 (± 1.63)	2.18 (± 0.16)
	WT	22.1 (± 1.88)	26.6 (± 0.96)	2.38 (± 0.17)
T1	GM	23.3 (± 1.80)	25.9 (± 1.10)	2.28 (± 0.20)
	WT	22.2 (± 1.81)	26.6 (± 0.95)	2.34 (± 0.19)
T2	GM	23.3 (± 1.85)	25.4 (± 0.69)	2.18 (± 0.13)
	WT	22.2 (± 1.81)	24.7 (± 1.05)	2.24 (± 0.120)

Supplementary Figure S1. Comparative phenotypic analyses between GM and WT plants. **A.** Phenotypic comparison between 60-day-old GM- and wild-type (WT) plants. **B.** Table of bud production 40 days after flowering in T0, T1, and T2. Number of seeds per cotton boll. Fiber weight per boll of different generations (Five bolls from 10 plants in each generation were used for data on weight and number of seeds). WT: wild-type plants (untransformed); GM: cotton genetically modified. Bars indicate standard error (SE, 95% confidence).

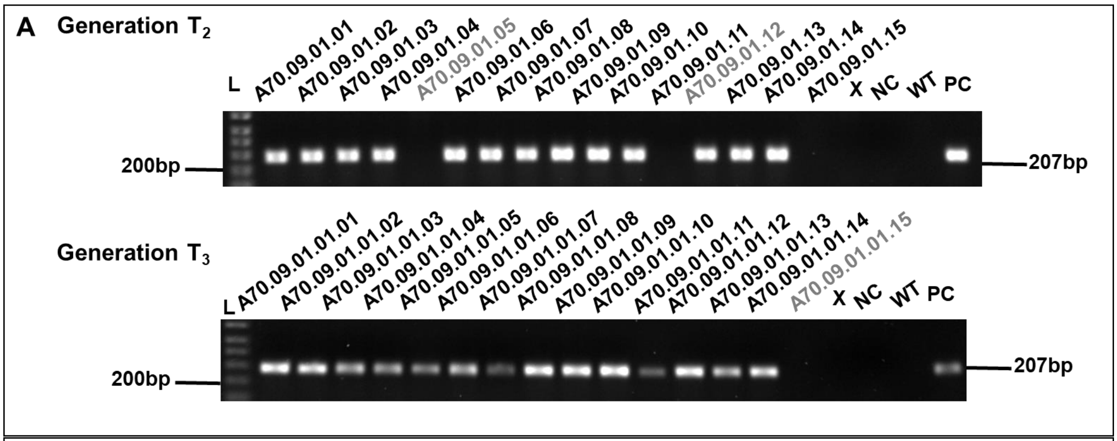

B

#Plant ID	Generation	Number of Plants	Number Plants PCR Positive	% Segregation
**A70.09.01**	T_2	15	13	86.6
**A71.41.01**	T_2	15	12	80.0
**A71.48.01**	T_2	15	11	73.3
**A73.22.01**	T_2	15	12	80.0
**A70.09.01.01**	T_3	15	14	93.3
**A71.41.01.01**	T_3	15	13	86.3
**A71.48.01.01**	T_3	15	14	93.3
**A73.22.01.01**	T_3	15	13	86.3

Supplementary Figure S2. Confirmation of cotton plant transformation in different generations through molecular analysis. A. Detection of the dsRNA fragment in GM cotton plants via PCR. Genomic DNA was extracted from leaves of T2 and T3 selected plants for amplification of the gene of interest (207 bp). **B.** Segregation transgene analyses on T2 and T3 generation cotton plants. Legend – Black letters: positive plants; Gray letters: negative plants; PC: positive control (vector with transformation cassette); WT: wild-type plants; L: 1.0 kb ladder; NC: negative control (ultrapure water). The X represents an empty well.

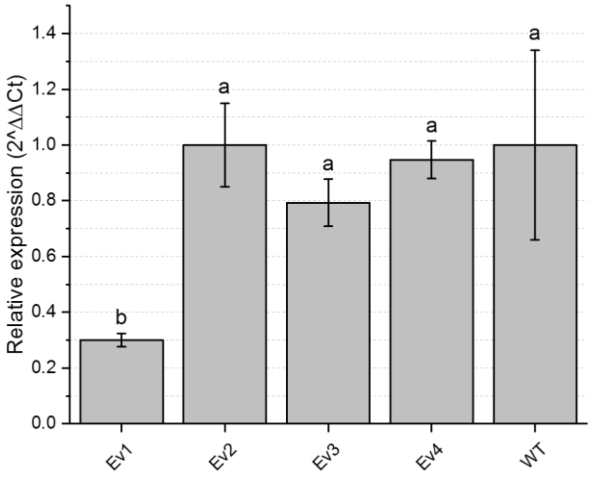

Supplementary Figure S3. Relative expression of CHS2 in CBW females fed GM cotton plants expressing dsRNA molecules after emerging from buds, 30 days after oviposition. Individuals fed on transgenic plants from different events (Ev) are compared with insects fed on wild-type cotton (WT). Lowercase letters indicate significant differences between treatments (t test, bonferroni corrected; p value < 0.05).

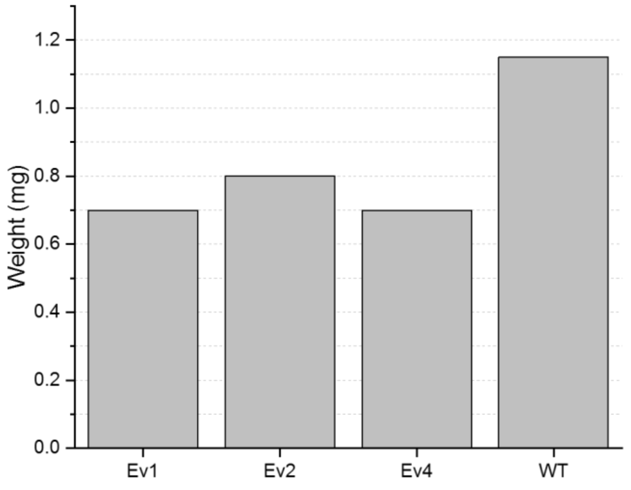

Supplementary Figure S4. Effects of dsRNA-expressing cotton plants in *A. grandis* larvae. The mean weight of larvae was determined by measuring a pool of 15 first instar larvae and dividing the total value by the number of individuals.

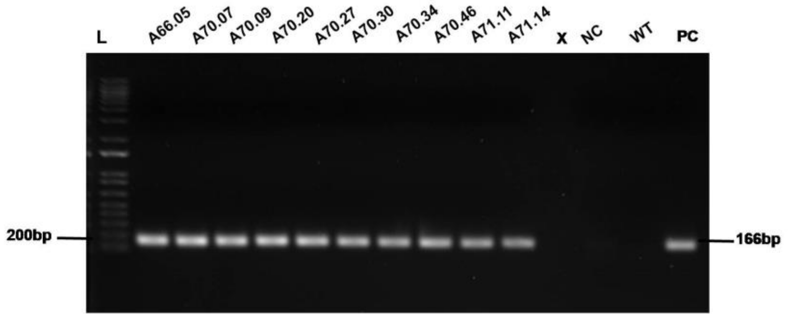

Supplementary Figure S5. Original image of the agarose gel 1% shown in figure 3A (first line). Black letters: positive plants; PC: positive control (vector with transformation cassette); WT: wild-type plants; M: 1.0 kb ladder; NC: negative control (ultrapure water). The X represents an empty well.

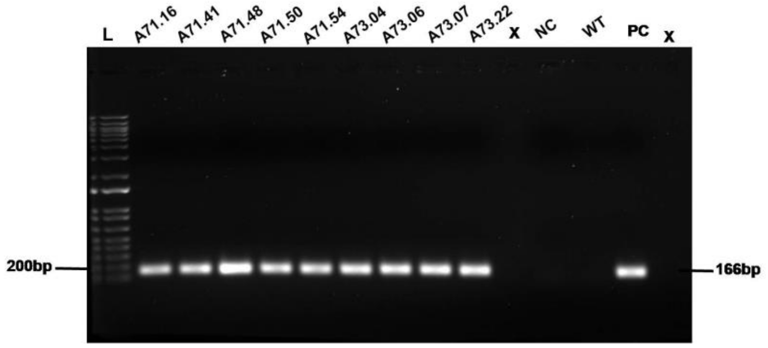

Supplementary Figure S6. Original image of the agarose gel 1% shown in figure 3A (second line). Black letters: positive plants; PC: positive control (vector with transformation cassette); WT: wild-type plants; M: 1.0 kb ladder; NC: negative control (ultrapure water). The X represents an empty well.

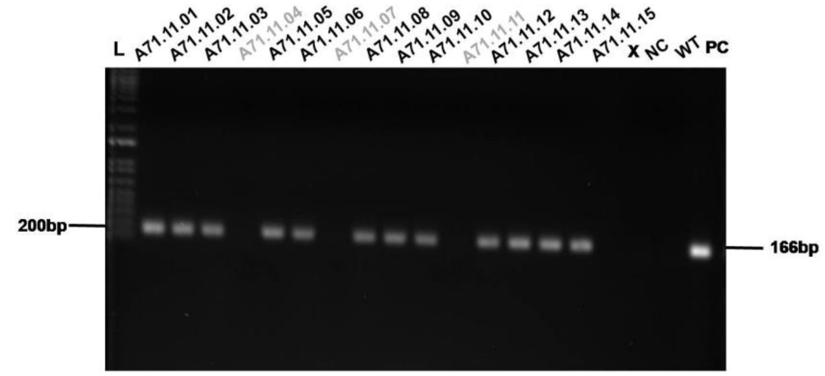

Supplementary Figure S7. Original image of the agarose gel 1% shown in figure 3B (first line). Black letters: positive plants; Gray letters: negative plants; CP: positive control (vector with transformation cassette); WT: wild-type plants; M: 1.0 kb ladder; CN: negative control (ultrapure water). The X represents an empty well.

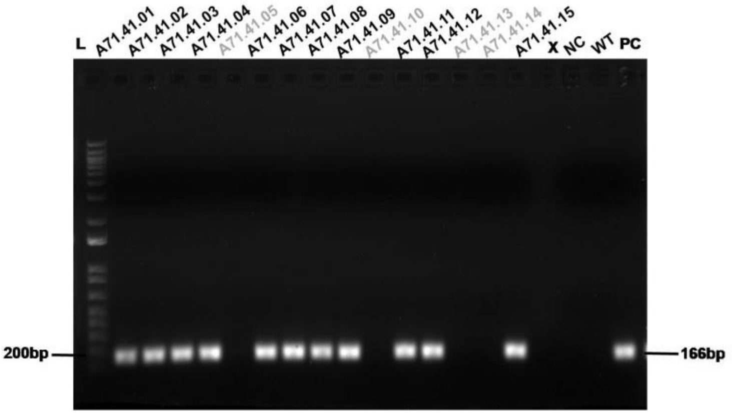

Supplementary Figure S8. Original image of the agarose gel 1% shown in figure 3B (second line). Black letters: positive plants; Gray letters: negative plants; PC: positive control (vector with transformation cassette); WT: wild-type plants; M: 1.0 kb ladder; NC: negative control (ultrapure water). The X represents an empty well.

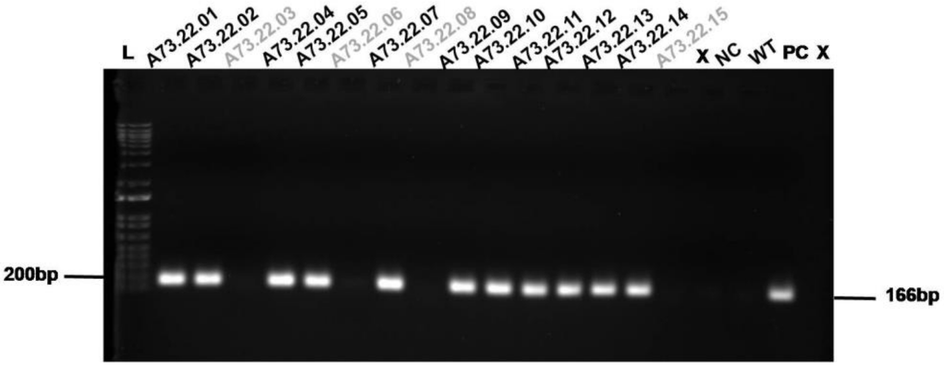

Supplementary Figure S9. Original image of the agarose gel 1% shown in figure 3B (third line). Black letters: positive plants; Gray letters: negative plants; PC: positive control (vector with transformation cassette); WT: wild-type plants; M: 1.0 kb ladder; NC: negative control (ultrapure water). The X represents an empty well.

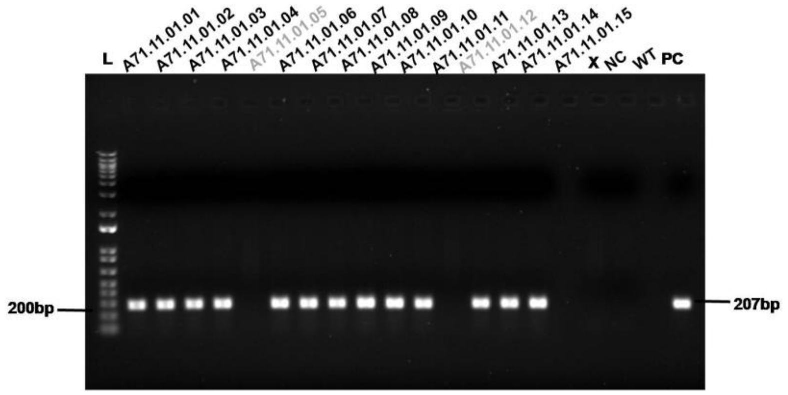

Supplementary Figure S10. Original image of the agarose gel 1% shown in figure S1A (first line). Black letters: positive plants; Gray letters: negative plants; PC: positive control (vector with transformation cassette); WT: wild-type plants; M: 1.0 kb ladder; NC: negative control (ultrapure water). The X represents an empty well.

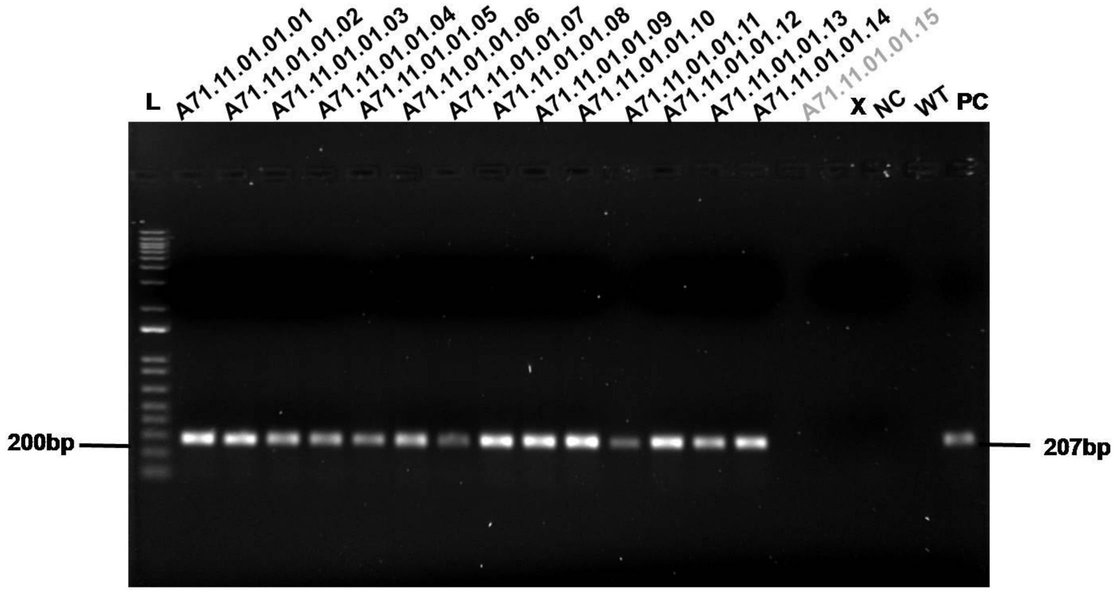

Supplementary Figure S11. Original image of the agarose gel 1% shown in figure S1A (second line). Black letters: positive plants; Gray letters: positive plants; PC: positive control (vector with transformation cassette); WT: wild-type plants; M: 1.0 kb ladder; NC: negative control. The X represents an empty well.
